# Antioxidant and hypoglycemic potential of phytogenic cerium oxide nanoparticles

**DOI:** 10.1038/s41598-023-31498-8

**Published:** 2023-03-18

**Authors:** Maarij Khan, Sohail, Naveed Iqbal Raja, Muhammad Javaid Asad, Zia-ur-Rehman Mashwani

**Affiliations:** 1 Department of Botany, Pir Mehr Ali Shah (PMAS)-Arid Agriculture University, Rawalpindi, Pakistan; 2 Institute of Biology/Plant Physiology, Humboldt-Universität Zü Berlin, Berlin, Germany; 3 University Institute of Biochemistry and Biotechnology (UIBB), PMAS Arid Agriculture University, Rawalpindi, Pakistan

**Keywords:** Biochemistry, Biological techniques

## Abstract

Plants provide humans with more than just food and shelter; they are also a major source of medications. The purpose of this research was to investigate the antioxidant and hypoglycemic potential of green synthesized CeONPs using *Mentha royleana* leaves extract. The morphological and physicochemical features of CeONPs were evaluated by UV–Visible spectrophotometry, Scanning Electron Microscopy, Energy Dispersive X-rays and Fourier-transform infrared spectrometry, Dynamic light scattering, Atomic Force Microscopy, Zeta Potential. The average size range of synthesized CeONPs diameter between 46 and 56 nm, crystalline in shape, with Polydispersity index value of 0.2 and subatomic particles mean diameter was 4.5–9.1 nm. The antioxidant capability of CeONPs was assessed using DPPH, ABTS^+^, hydrogen peroxide, hydroxyl radical scavenging, and reducing power tests. The hypoglycemic potential of CeONPs was investigated using alpha-amylase, alpha-glucosidase, glucose absorption by yeast cells, and antisucrase. The effective concentrations were 500 and 1000 µg/ml found good in suppressing radical species. To explore the hypoglycemic potential of CeONPs, alpha-amylase, alpha-glucosidase, glucose absorption by yeast cell, and antisucrase assays were performed. Glucose absorb by yeast cells assay was tested for three distinct glucose concentrations: 5 mmol/L, 10 mmol/L, and 25 mmol/L. Green synthesize CeONPs showed a dose-dependent response, higher concentrations of CeONPs imposed a stronger inhibitory impact on the catalytic site of enzymes. This study suggest that CeONPs could possibly binds to the charge carrying species and act as competitive inhibitor which slow down the enzyme substrate reaction and prevents enzymatic degradation. The study’s findings were outstanding, which bodes well for future medicinal applications of CeONPs.

## Introduction

The plants themselves are like little factories producing phytochemicals. In nature, there is a plethora of phytometabolites that are toxic-reducing^1^. It is possible to produce nanoparticles by reducing bulk salts into nanostructures utilizing the reducing power of plant extract. In order to reduce the toxicity of the chemical, the NPs are coated with a significant number of phytochemicals on their surfaces^2^. Many methods, including physical, chemical, and biological ones, are now in use for the synthesis of NPs. The physical and chemical methods are completely chemical based techniques, costly, and special equipment’s are required to control conditions for NPs preparation^3^. NPs can be synthesized using algae, fungus, bacteria, and plants or their derivatives. In the given study we prefer the plants to synthesize NPs because plants are more cost-effective, largely and easily available organisms, and less control conditions are required for storage, fewer chances of contamination^4^. The CeONPs have wide applications in engineering and biological sectors, such as high-temperature oxidation protection materials, potential pharmacological compound, solid-oxide fuel cells, solar cells, and catalytic materials, heterogeneous catalytic reactions. CeONPs are popular due to their unique surface charge chemistry and 3D position to interact with others molecules, compounds and living tissues. During industrial processes CeONPs are utilized as active chemical component for oxidative coupling of methane, automobile exhaust-gas treatments, and water–gas shift reaction. Recent researches reported that CeONPs display multi-enzymes properties including peroxidase, catalase, superoxide dismutase mimetic properties and emerged as a lucrative and fascinating material in biological fields like biomedicines, drug delivery, bioanalysis, and bioscaffolding^5^. ROS generation is a significant mechanism in immune system and become active in case of nay infection. The ROS generation play an important role in cell signaling but the excessive and misdirected generation of ROS responsible for inflammatory disorders such as cancer, diabetes, cardiovascular disorder, and neurodegenerative diseases. The antioxidant scavenging potential of CeONPs in natural environment mimic multi-enzymatic catalytic activities and rapidly bind with the free radicals in the natural environment. The electronic configuration of cerium is interconverting between Ce^+3^ and Ce^+4^. The surface grooves at CeONPs surface exhibit electropositive charge and efficiently interact with free charge carrying species and reduce oxidative stress inside the body^6^.

In the present study, CeONPs were synthesized by using *Mentha royleana* leaves extract*.* The *Mentha royleana* belongs to the family Lamiaceae and is a wild species of Mint. The *Mentha royleana* is commonly called wild mint and found in cold and hilly areas of Pakistan^7,8^. All *Lamiaceae* family members possess scented leaves because they store unique phytochemicals combinations. The phytochemicals possess magical medicinal capabilities^9^. In previous times mint leaves were used as carminative, antiulcer, antidiabetic, antiseptic, and anti-inflammatory agents^10,11^. The *Mentha royleana* phytochemical*s* profile consists of volatile components, essentials oils, hydrocarbons, and phenolic compounds, and flavonoids, metabolites. The *Mentha royleana* linalyl acetate and linalool volatile components, essentials oils profile including menthol, carvone, and menthone, linalool, metabolites composition of *Mentha royleana* menthol, menthone, 1,8-cineole, 3-octyl acetate, beta-caryophyllene, carvone,1,2-epoxyneomenthyl acetate, 3-octanol, 3-octanone, geraniol, decyl acetate, caryophyllene oxide, elemol, limonene, isomenthone, phenolic components of *Mentha royleana* included rosmarinic acid, chlorogenic, and caffeic acid. Flavonoids 5-hydroxy-6,7,3′,4′-tetramethoxyflavone, methylated luteolin-glucuronide, Eriodictyolglucopyranosyl-rhamnopyranoside, and luteolin-glucuronide.

Recent advances in the synthesis of NPs have increased their applications in a variety of fields, making nanotechnology an increasingly popular scientific topic. The NPs are small miniatures, are aggregates of atoms and molecules, and possess a high surface-to-volume ratio and high absorption power. The physicochemical properties differentiate NPs from bulk salt^12^. Cerium is a ductile metal that belongs to the lanthanide category in the periodic table. Cerium captured researchers’ attention due to its unique oxidation state^13^. The cerium salt exhibits an eccentric electronic configuration [Xe]4*f*^1^5*d*^1^6*s*^2^ which is found in few metals in the periodic table^12^. The distinguishing features make cerium metal enchanting and charming in various industries including pharmacy, agriculture, medical, electrical, and chemicals. The peculiar and iconic oxidation state makes CeONPs matchless with other metallic NPs. The CeONPs contain positively charge holes on their surface due to vacancy in d” and f” orbitals. Sometimes electrons in s” orbital jump from lower oxidation state to higher then CeONPs show CeO_3_ reduced form^13,14^. The electropositive charge surface magnetizes CeONPs antioxidant nature. The CeONPs bind free radicle species hydroxyl radical, hydrogen peroxide, superoxide anion radical, and singlet oxygen effectively and reduced oxidative stress. The antioxidant nature makes CeONPs an effective agent against oxidative stress induced diseases^15–18^.

Glucose is the primary source of metabolic energy approximately for all living organisms. Glycolysis is an essential cellular process for ATP (Adenosine triphosphate) generation (Ajila et al., 2008). Diabetes is caused by a chronically elevated blood sugar level and a decreased generation of insulin. Due to damage to pancreatic cells, insulin production decrease, and glucose is never absorbed inside the cells and continuously accumulates in the blood. The high blood glucose level deteriorates the other function of the body. in the given study CeONPs were checked to reduce oxidative stress and to reduce the activity of enzymes involved in the digestion of polysaccharides^19^. Diabetes is a world-leading metabolic disorder. Around 10.5% of the global adult population suffered from diabetes in 2021—by the year 2045, this number is expected to rise to over 12%^20^. Diabetes is included among top ten leading causes of deaths^15^. Type-2-diabetes arise due to constant intake of high glucose or sometimes due to degradation of insulin producing pancreatic cells. Diabetes increase the chances of cardiovascular diseases, joint swelling, chronic kidney disorders, uncontrolled urination, and heart attack^15^. More than 95% of diabetic patients are affected with type-2-diabetes and the remaining 5% with type-1-diabetes (characterized by genetic abnormalities in insulin-producing cells)^21^. According to WHO (World Health Organization) previous data in 2014, 8.5% of adults aged 18 and more had diabetes. In 2019, the death toll from diabetes rose to 1.5 million, accounting for 48% of all diabetes-related deaths, with a median age of about 70 years^20^.

The goal of this study was the green synthesis of CeONPs by using *Mentha royleana* leaves extract which makes CeONPs highly biocompatible and biodegradable for biological systems. The mechanism of CeONPs synthesis involve three basic steps (1) the ionization of cerium nitrate hexahydrate salt in deionized water (2) the reduction of the cerium nitrate hexahydrate with plant extract and this is the stage at which cerium (Ce) separate from nitrate (NO_3_) 3) and finally CeONPs encapsulated and coated with various phytochemicals of the plant extract. The second goal was the exploration of antioxidant potential of green synthesis CeONPs in presence of different radical’s species. And the third most crucial target was the determination of the hypoglycemic potential of green synthesis CeONPs by inhibiting the catalytic activity of enzymes involved in the digestion of disaccharides and polysaccharides to lower the glucose release in the body. CeONPs act as a competitive inhibitor in the enzymatic reaction and perform the antienzyme role. This declines the enzyme–substrate reaction and prevents the enzymatic breakdown^22^. CeONPs have the possibility of functioning as substrate analogs and competitive inhibitors and CeONPs are unable to metabolize in enzyme-catalyzed reactions. The electropositive charge on the CeONPs surface promotes its activity to form a bond between the active site and CeONPs^23^. The results of all *in-vitro* activities flaunt that green synthesized CeONPs are good antioxidants and contain marvelous antienzymes potential. Green synthesis CeONPs can be used for *in-vivo* studies and pharmacological trials based on the results of this investigation.

## Materials and methodology

### Preparation of leaves extract and CeONPs

**Figure 1:**
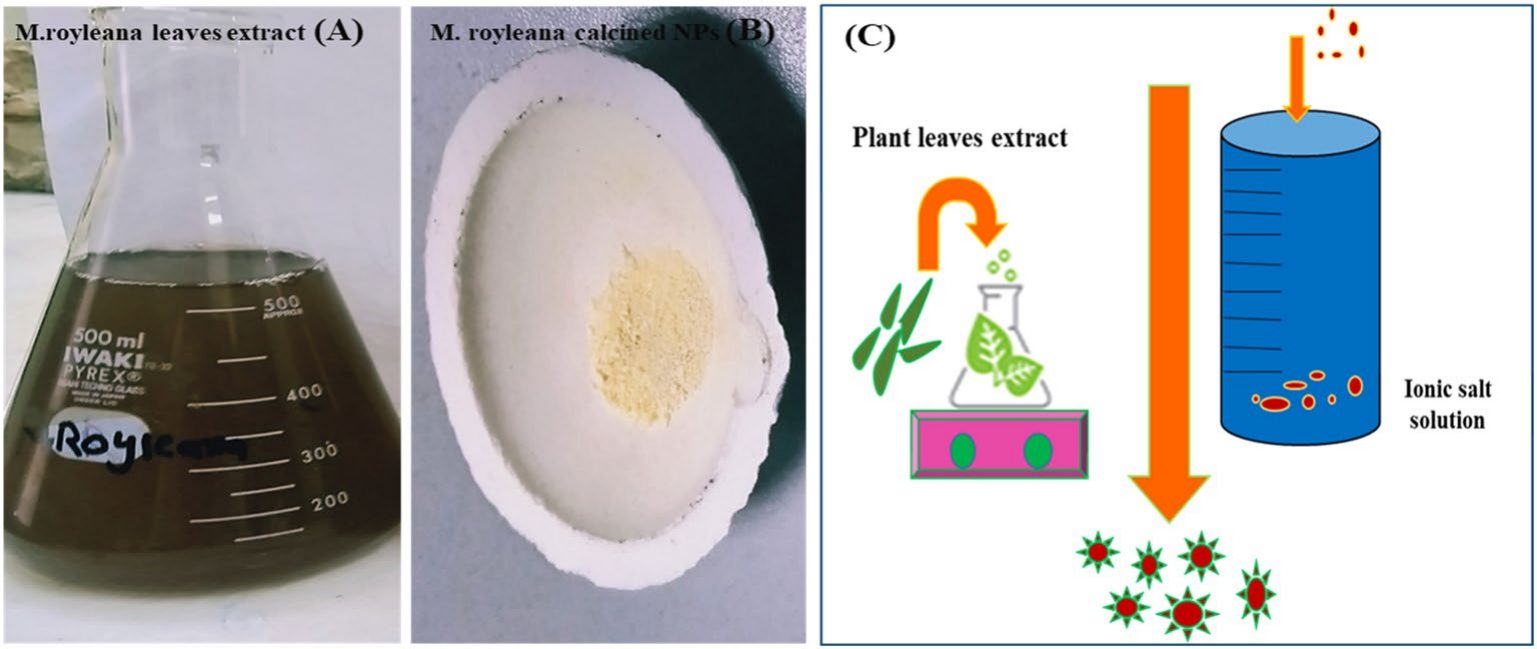
(**A**) Aqueous extract of *Mentha royleana*, (**B**) Calcined CeONPs at 400 C^0^ for 2 h, (**C**) Schematic representation of the general process of green synthesized CeONPs synthesis.

The *Mentha royleana* was collected in Upper Dir (Latitude: 35° 12′ 20.99″ N Longitude: 71° 52′ 32.02″ E) District of Khyber Pakhtunkhwa, Pakistan. To make extract, 10 g of dried *Mentha royleana* leaves powder was mixed with 120 ml of deionized water and heated to 100 °C on a hot plate covered with aluminum foil for 2 h (Fig. 1A). The extract was filtered by Whatman no.1 filter paper. For the green synthesized CeONPs, 0.04 g salt of Ce (NO_3_)_3_.6H_2_O was dissolved with 400 ml of distilling water and left in the solution for 40 min on a hotplate at 150 °C^24^. The next step was to add the 75 ml of plant extract to the salt solution drop by drop. The solution was kept on a hotplate for 4 h. The synthesis of CeONPs was confirmed by the brown color precipitate in the solution. Remove the solution from the hotplate. Left the solution overnight, after overnight treatment transfer solution on the hotplate again for 2hand finally centrifuge CeONPs solution and washed 3 to 4 times with concentrated methanol. Transfer CeONPs in the oven at 64 °C for 12 h (Fig. 1C). The purified green synthesized CeONPs were calcined in a muffle furnace at 400 °C for 2 h (Fig. 1B)^25^.

### Morphological and optical characterization

To evaluate the size, shape and chemical nature of green synthesized CeONPs, different characterization technique has been used in this study^26^. The scanning electron microscopy (SEM) was carried out to collect micrographic images^1^. Energy depressive X-Rays were used to reveal the chemical composition of green synthesized CeONPs while chemical groups attached to the surface of CeONPs were analyze by using Fourier transform infrared spectroscopy (FTIR)^12^. The principle of Dynamic Light Scattering (DLS) is based on the Brownian movement of suspended particles in water. When particles in a solution are uniformly dispersed, they travel in all directions, and solute particles constantly collide with solvent molecules.

### Antioxidant potential evaluation of CeONPs

#### Antioxidant DPPH (2,2-diphenyl-1-picrylhydrazyl) Assay

To determine the antioxidant capacity of phytofabricated CeONPs, the protocol developed by^7^ was used. Different concentration of CeONPs (62.5, 125, 250, 500, and 1000 µg/ml) were investigated for the antioxidant potential. The reaction mixture contained 1 ml of 2,2-diphenyl-1-picrylhydrazyl (DPPH) and 1 ml of different concentration of CeONPs. The mixture was shaken vigorously and incubated at room temperature for 30 min, and the absorbance was measured at 517 nm. Except for phytofabricated CeONPs, all compounds were present in the control sample, while ascorbic acid was used as a standard reagent. The final percentage of inhibition was calculated as follows:

$${\text{Antioxidant Percentage }}\% \, = \, \left[ {\left( {{\text{A}}_{0} {-}{\text{ A}}_{{1}} } \right)/{\text{A}}_{0} } \right] \, \times { 1}00$$

where: A_0_ = Absorbance of control, A_1_ = Absorbance of treatments.

#### ABTS (2,2'-azino-bis (3-ethylbenzothiazoline-6-sulphonic acid) antioxidant assay

The ABTS^+^ radical scavenging assay of green synthesized CeONPs was performed as per the stated protocol^25^. The reaction mixture was prepared by mixing 1 ml CeONPs of different concentrations (62.5, 125, 250, 500, 1000 µg/ml), and 1 ml of ABTS^+^ in K_2_S_2_O_8_. The methanol (1 ml) used as a negative control and ascorbic acid as a positive control. The absorbance was measured at 734 nm after 6 min the percentage of inhibition.

#### Hydrogen peroxide antioxidant assay

To assess the hydrogen peroxide assay Yakoob et et al.^7^ method was used. The reaction mixture contained 100 µl of CeONPs, 300 µl phosphate buffer (50 mM, pH 7.4) and 600 µl hydrogen peroxide (2 mM H_2_O_2_ in phosphate buffer, 50 mM, pH, 7.4). The mixture was shaken vigorously for 10 min and the absorbance was measured at 230 nm by using spectrophotometer (Model U-2900). Ascorbic acid was used as standard and phosphate buffer as blank solution.

#### Hydroxyl radical antioxidant assay

The hydroxyl radical assay was carried out in accordance with the published protocol of^21^. The reaction mixture contained of 750 µl phytofabricated CeONPs, 45 µl Sodium phosphate buffer (200 mM, pH 7.0), 15 µl Deoxyribose (10 mM), 150 µl FeSO_4_ and EDTA (10 mM), 15 µl H_2_O_2_ (10 mM) and 525 µl deionized water. The solution was kept on incubator for 4 h. The reaction was stopped by the addition of (2.8%) 75 µl trichloroacetic acid and 75 µl TBA (1% in 50 mM NaOH). The mixture was then kept on boiling water bath for 10 min. The absorbance was recorded at 520 nm by using a UV–visible spectrophotometer (Model U-2900). Methanol was employed as a blank and ascorbic acid as a standard sample.

#### Reducing power assay

Reducing power assay of green synthesized CeONPs were determined by method of Pavithra et al.^27^. About 100 µl of different concentration of green synthesized CeONPs (62.5, 125, 250, 500, 1000 µg/mL) were mixed with 2.5 mL of (1%) potassium ferricyanide and 250 µl of 0.2 mol/L sodium phosphate buffer. The mixture was then incubated at 50 °C for 30 min. The reaction was terminated by adding 2.5 mL of 10% trichloroacetic acid following centrifugation at 3000 rpm for 10 min. The upper pellet of the centrifuged sample was mixed with 0.5 mL of 0.1% ferric chloride and 2.5 ml of de-ionized water. The absorbance of the sample was noted as 700 nm using spectrophotometer (Model U-2900).

### Hypoglycemic activities

#### CeONPs effect on glucose uptake by yeast cells

To check glucose uptake, 5 µg of yeast was dissolved in 1 ml of deionized water, vortexed for 10–15 min, following centrifugation at 21,000 rpm for 5 min^7^. In deionized water, 10% (v/v) concentration of yeast suspension was prepared. About 100 µl of different concentration of green synthesized CeONPs (62.5, 125, 250, 500, 1000 µg/mL) were mixed with 1 ml of glucose solution (5, 10, 25 mmol/L), followed by incubation at 37 °C for 10 min ^o^C. To begin the reaction, 100 μl of yeast suspension was combined and vortexed before incubating at 37 °C for 60 min. The reaction mixture was centrifuged at 3800 rpm for 5 min, and the glucose concentration was measured at 540 nm. The following formula was used to calculate the percentage of glucose uptake by yeast cells:

$${\text{Hypoglycemic }}\% \, = \, \left( {{\text{Abs control }}{-}{\text{ Abs sample }}/{\text{ Abs control}}} \right) \, \times { 1}00$$

#### Alpha-amylase inhibition assay

The methodology developed by^27^ was used to calculate the Alpha-amylase inhibition assays. In DMSO, different CeONPs concentrations of 62.5, 125, 250, 500, and 1000 g/ml were prepared. The reaction mixture was prepared by dissolving 3 mg of Alpha-amylase in 20 mM phosphate solution (pH 6.7) containing 6.5 mM sodium chloride. About 250 µl from the reaction mixture were added to various concentrations of green synthesized CeONPs followed by incubation at 37 ^0^C for 30 min. After pre incubation, 250 µl of 0.5% starch solution in 20 mM phosphate buffer pH 6.9 was added. The reaction mixture was then incubated 37 °C for 15 min. The reaction was stopped with 2 ml of 96 mM 3, 5 dinitrosalicylic acid colour reagent. The micro plate was then incubated in a boiling water bath for 5 min and cooled to room temperature. The absorbance of the sample was measured at 540 nm using a UV–visible spectrophotometer (Model U-2900) with glucobay as the standard drug.

#### Alpha-glycosidase inhibition assay

Alpha-glucosidase assay was assisted by using the methodology of^28^. Approximately 5 mg of enzyme powder was dissolved in 1 ml of maleate buffer for preparation of serial dilutions. The enzymatic reaction was started by adding 200 μL of the substrate (p-nitrophenyl-a-D-glucopyranoside 2 mmol) following incubation at 37 °C for 30 min. The reaction was then stopped by standing the test tube in boiling water for 5 min. The reaction mixture was equipped with 100 µl of disodium hydrogen phosphate (0.1 M), and the absorbance of the liberated p-nitrophenol was recorded at 400 nm using a spectrophotometer (Model U-2900) while glucobay was utilized as a standard drug.

#### Sucrase inhibition assay

In order to assess the sucrase inhibition assay, 10 µl of crude enzyme solution, 10 µl of different concentration of CeONPs (62.5, 125, 250, 500, 1000 µg/ml), were mixed with 100 µL of maleate buffer following incubation at 37 °C for 10 min. The reaction was started by the addition of substrate 100 µl (60 mmol of sucrose) and then incubation at 37 °C for 30 min in water bath. The reaction was stopped by immersing glass tubes in boiling water bath for 10 min. The released glucose was measured with a glucometer. The control was taken with containing CeONPs while glucobay was utilized as the standard drug^21^.

### Statistical analysis

All experiments were done with three biological replicates. The data were statistically processed using SPSS 20 for ANOVA, and the mean significant differences were separated using Duncan's Multiple Range Test (DMRT).

### Methods

We confirmed that all methods were performed in accordance with the relevant guidelines and regulations.

### Research involving plants

The fresh leaf material used for the greens synthesis of NPs in the study were purchased from the National Agriculture Research Center Islamabad with prior permission to use for research purposes.

## Results and discussion

### Characterization of CeONPs

**Figure 2:**
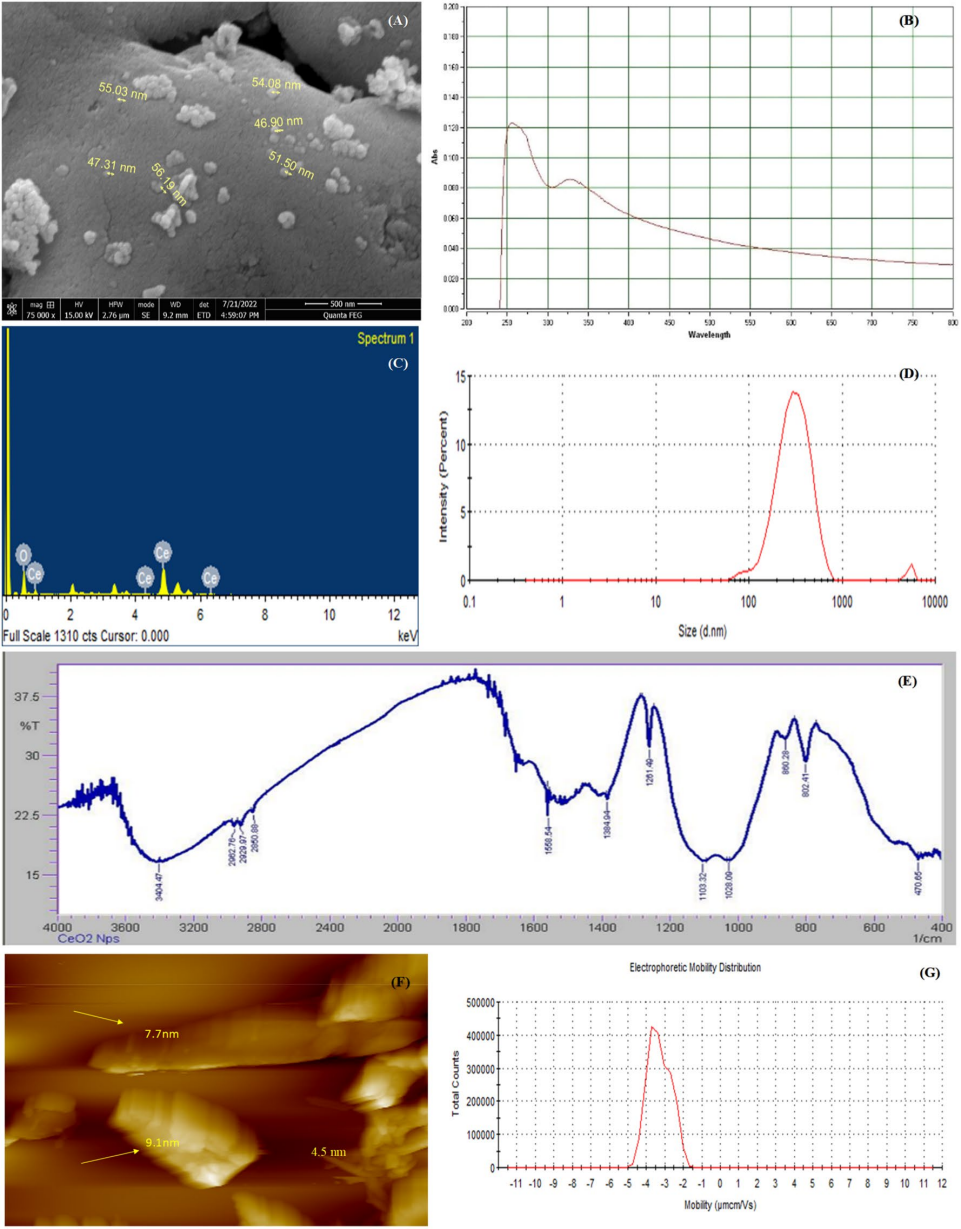
(**A**) SEM image of green synthesized CeONPs with 46 to 56 nm size (**B**) UV–Visible spectrometry image of green synthesized CeONPs peak at 260 nm (**C**) EDX image of green synthesized CeONPs confirm the presence of Cerium (**D**) DLS image of green synthesized CeONPs which show size less the 100 d.nm (**E**) The peaks at FTIR image of green synthesized CeONPs express the presence of compounds on NPs surface. (**F**) Atomic Force Microscopy observe nanoparticles magnitude (size) of CeONPs ^M.R^ 4.5–9.1. (**G**) Zeta potential measurement of the effective electric charge on the NPs surface and the electro mobility was -3.293 mV depicts the positive charge at CeONPs ^M.R^.

In order to confirm the synthesis and the catalytic activity of CeONPs, some important parameters are necessary to check like average particles size, radius, chemical nature, and surface properties of CeONPs. The SEM image revealed that CeONPs average particle size 46 nm to 56 nm (Fig. 2A). A considerable size stub was chosen for the SEM, and double-sided tape conductive carbon tape was adhered to the stub after dispersing 1 µg of CeONPs in methanol and sonication for 30 min. Then attached a substrate on a double-sided conductive carbon tape and placed a drop of CeONPs on the substrate surface and let them dry. The store stub was used to explore the size of CeONPs at various magnifications^12^. UV–Visible results confirmed the presence of CeONPs in the sample showing a wide peak at 337 nm, a surface plasma resonance (SPR) band characteristic of CeONPs was found (Fig. 2B). The EDX results authenticate the presence of cerium in the sample. The peaks of (Ce) attest to the presence of cerium salt that was utilized for CeONPs green synthesis (Fig. 2C)^12^. For the DLS CeONPs were suspended in Milli-Q ultrapure water and fabricated using a probe sonicater (Fisher Scientific Model FB120). The Polydispersity index value measured was 0.2 and the Polydispersity index width was 146 d. nm indicating the homogeneity of sampled solution. The DLS image is generated mostly by the Brownian movement of the solute in the solution. The uniformity of the suspended particles is required for pharmaceutical drug material applications. (Fig. 2D)^7^. The FTIR peaks and stretching bands, which are common between three CeONPs, –CH (Alkene) at 2962 cm^−1^ and –CH (Alkyl) at 3404.47 cm^−1^. The wavelength 2850.88 and 2929.97 cm^−1^ indicated the presence of alkene (C = C) group. 1558.54 cm^− 1^ indicated the presence of Alpha–beta unsaturated ketone group C = C, O–H at 1384.94 cm^−1^ phenol and C–O at 1261.48 cm^−1^ indicate Alkyl-aryl-ether and C–O at 1103.32 cm^−1^ indicate the presence of aliphatic group C–O. 1028.09 cm^−1^ depicted the presence of Anhydride CO–O–CO. 860.28, 802.41 cm^−1^ depicted the occupation of C = C Alkene group. 470.65 cm^−1^ indicated the habitation of C-I bending belong to Halo compound. The FTIR spectral wavelength for CeONPs was ranged between 400 and 4000 nm (Fig. 2E)^12,12^. Zeta potential is a test to evaluate the electric charge on the NPs surface. The net charge on NPs surface is screened through the abundance of ions carrying the opposite charge near the NPs body. The loop of these opposite electric charges float along with NPs. Actually the zeta potential is a test to identify the difference between the charge in bulk fluid in which NPs are suspended and the charge on the loop of ions that encirculate the opposite charge NPs surface. The particles containing positive charge always bind with negative charge ions. Higher the electrostatic repulsion increase the stability of NPs in medium. Zeta potential was measured by using two gold electrodes that were added in the deionized water contained the dispersed NPs. When the voltage was applied the NPs always move towards the electrode possess the opposite charge. A Doppler method is used to evaluate the NPs velocity (speed) as function of voltage. A laser beam passes through the cell (contain solution of dispersed NPs), and laser pass through the NPs solution, the intensity of scattered light fluctuates at a frequency proportional to the NPs velocity (speed). The NPs velocity (speed) can measure at different voltages, and this data is used to calculate the zeta potential. The zeta potential of the NPs measures around or less than − 28 mV, confirm that the particles are agglomerated Fig. 2F. Various techniques are currently used to characterize NPs size. The calculation of exact NPs size is a necessary step to determine NPs physical interactions with biological material. AFM is consider an attractive tool for the characterization of NPs (2). The AFM resolution power is very high reaches about 0.1 nm, related with sample thickness, taping or non-contacting mode and applied voltage. AFM is a 3D digital way of measuring the NPs magnitude. CeO_2_NPs^M.R.^ magnitude was measured through AFM and average particles size was 4.5 to 9.1 nm, which was an efficient size to penetrate inside the organelles Fig. 2G.

### Antioxidant Activities

#### DPPH Assay

**Figure 3:**
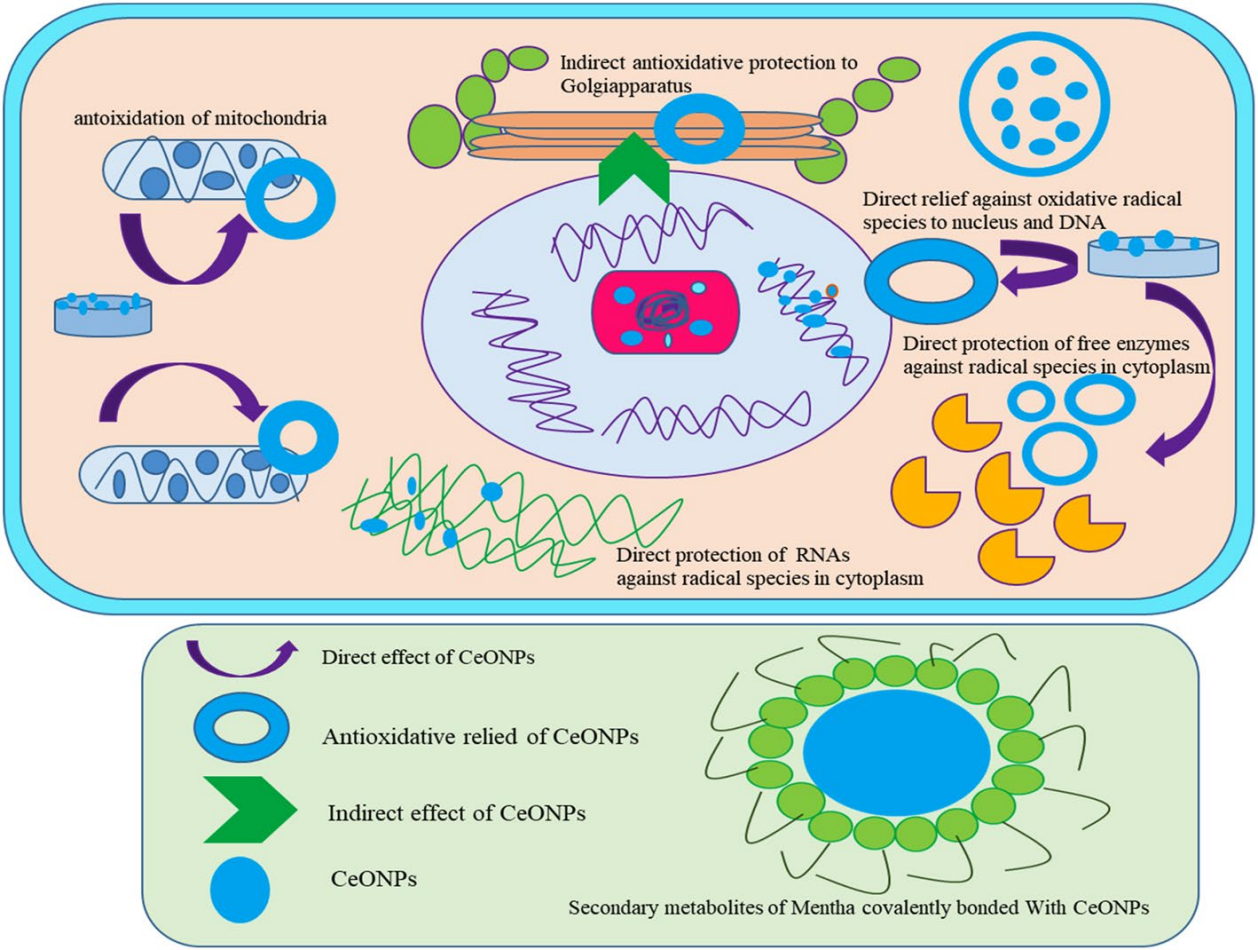
The antioxidant mechanisms of green synthesized CeONPs on numerous organelles within the cell are represented schematically. The CeONPs easily cross plasma membrane due to nano-size and binding with organelles directly or indirectly. The CeONPs possess efficient anti-oxidative strength. The direct and indirect reaction of CeONPs due to their electropositive nature binds free charge carrying species in the environment^53^. In this way, it directly integrates with free radical species. In the indirect response when one organelle produces ROS and its products are transferred to another organelle and reach another organelle nanosize CeONPs bind these ROS before arising disturbance. In this way, CeONPs are helpful to reduce direct and indirect oxidative stress^54^.

**Figure 4:**
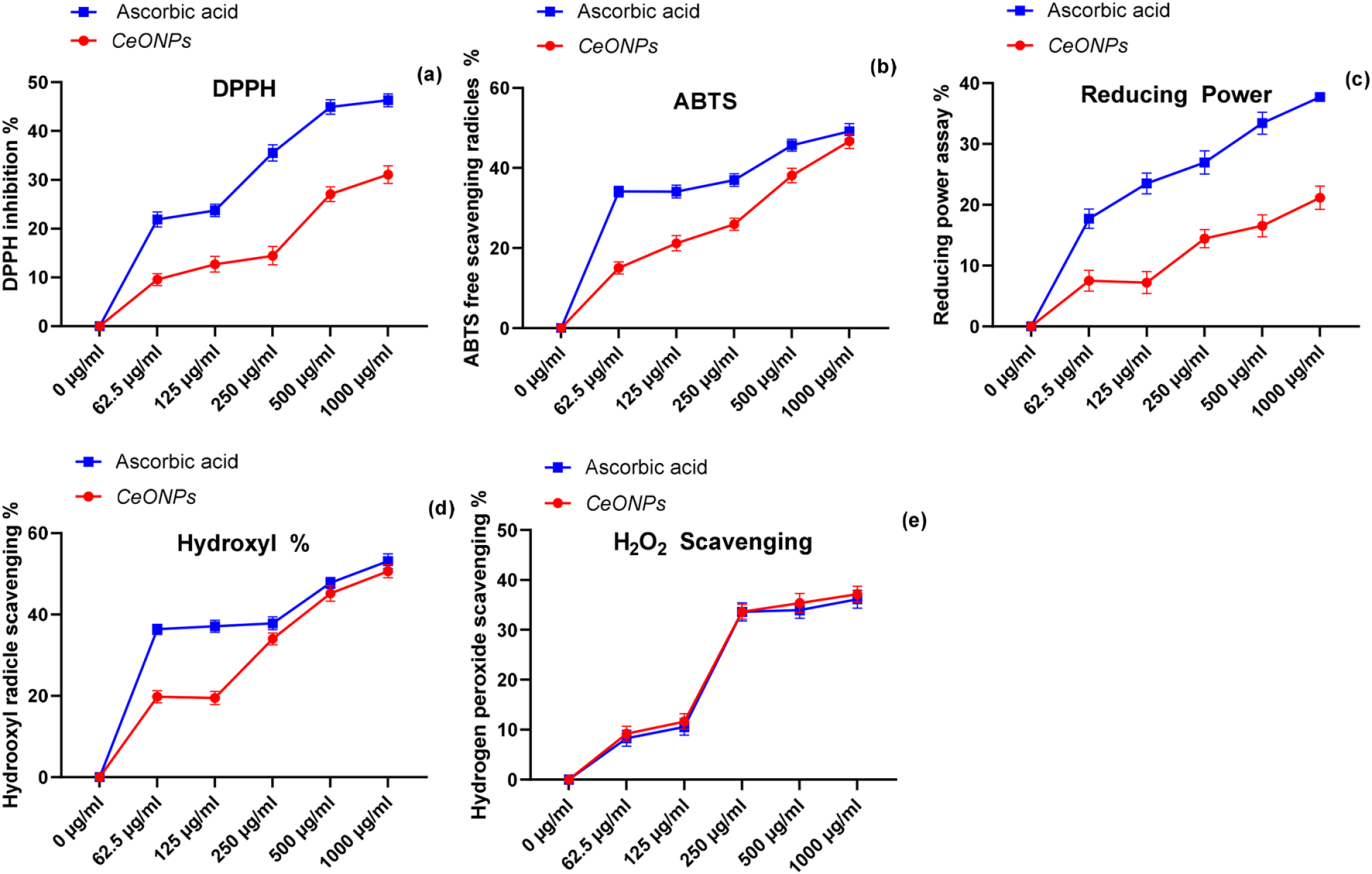
(**a**) Figure explain the DPPH radicals inhibition percentage of CeONPs was little low compared with standard ascorbic acid (**b**) Figure demonstrate the ABTS^+^ radicals scavenging potential of CeONPs show high antioxidant activity compared with ascorbic acid (**c**) Reducing power of CeONPs was slightly low form ascorbic acid (**d**) Hydroxyl radicles cleaving potential of CeONPs was higher than ascorbic acid (**e**) Figure exhibit the CeONPs quenching power was against hydrogen peroxide radicles in comparison with ascorbic acid The value of level of significance was *p* < 0.05 for all antioxidants activities. A *p*-value lesser than 0.05 was consider significant along with the post hoc test.

The DPPH antioxidant assay was used to assess the antioxidant capacity of green synthesized CeONPs against free radicals^29^. The imbalance between ROS formation and quenching by the endogenous antioxidant system disrupts physiological processes and is responsible for mutations in macromolecules such as DNA, RNA, proteins, and lipids, as well as accelerating cellular stress in cell signaling pathways^30–32^. ROS production is accelerated by the presence of high glucose concentration through various mechanisms including glucose auto-oxidation, oxidative phosphorylation, protein kinase C activation, glycation (non-enzymatic bonding of free reduced sugar with free amino acids like DNA, RNA) hexosamine metabolism, methylglyoxal formation (Fig. 3). Pancreas is crucial for the production of enzymes involved in the digestion of food and maneuver blood glucose level. Oxidation of pancreatic cells proceeds destabilization of the hormonal content of the pancreas and the result is high blood glucose level (hyperglycemia). In current experimental work, *Mentha royleana* mediated CeONPs were found effective in decrement of ROS levels. The green synthesized CeONPs show the maximum antioxidant activity of 31% and ascorbic acid of 46%. The inhibition percentage of green synthesized CeONPs is quite close to ascorbic acid (Fig. 4a). The medicinal plants deposit various essential metabolites which plant used against various biotic and abiotic stresses^32^. According to one study, the antioxidant potential of green synthesized CeONPs in DPPH solution rose as the concentration of CeONPs in the solution increased^33^. Another study discovered that the antioxidant potential of green synthesized CeONPs is greater than that of butylated hydroxytoluene. (BHT)^24^.

#### ABTS antioxidant assay

The radical scavenging capacity of green synthesized CeONPs underpins their ABTS^+^ scavenging activity. The ABTS^+^ assay assesses antioxidants' ability to scavenge oxidative species produced by ABTS + . The ABTS^+^ radicals are formed by a strong reaction between the ABTS^+^ salt and the highly oxidizing agent potassium persulfate. A decrease in absorbance is observed as antioxidant scavenging potential is increased. The blue/green ABTS^+^ solution turned pale yellow and then colorless. The IC_50_ value for ascorbic acid was 5.39 g/ml and the percentage was 49.7%, while the IC_50_ value for *Mentha royleana* was 5.57 g/ml, and the percentage scavenging potential of *Mentha royleana* mediated CeONPs was 46.7%, which is excellent for commercial applications (Fig. 4b). In a dose-dependent manner, the higher percentage of green synthesized CeONPs reduced the ABTS^+^ radical's species^25^. Other data suggest that CeONPs scavenge ROS preferentially from normal cells and protect normal cells against reactive oxygen species^34^. The findings of the given activity revealed that *Mentha royleana*, a wild mint species, has unique and useful phytochemicals with higher antioxidant potential than others.

#### Hydrogen peroxide scavenging assay

Hydrogen peroxide generates hydroxyl radicals that cause lipid peroxidation in exposed cells, resulting in DNA damage and cell death. Acute exposure to hydrogen peroxide is hazardous to one's health. The hydrogen peroxide irritates the skin at the point of contact. The mitochondria are organelles that are produced by a specific enzyme that governs cell development and death. However, the enzyme that decomposes hydrogen peroxide before it converts into hydroxyl radicals is already present in the cell^35^. Literally, hydrogen peroxide synthesis in the body cells is a pursuit to protect the body from even more dangerous substances like superoxide radicles but when a living body facing disease conditions, the function of these enzymes declines then the hydrogen peroxide level increase from its limits, and scavenging potential is reduced and cause lipid peroxidation. In the current study, the *Mentha royleana*-mediated CeONPs have been discovered to be particularly effective at lowering the level of hydrogen peroxide in cells. The green synthesized CeONPs scavenging potential was 8% and (IC_50_ 28.89), indicating weak activity when compared to the ascorbic acid antioxidant potential of 30% and (IC_50_ 6.98), which is greater than CeONPs (Fig. 4e). Another study compared benzoate derivatives to cinnamate analogs (organic nature) and discovered that benzoate derivatives were more powerful at quenching hydrogen peroxide reactive species^36^.

$${\text{H}}_{{2}} {\text{O}}_{{2}} {\text{scavenging activity percentage }} = \, \left[ {\left( {{\text{A}}_{0} {-}{\text{ A}}_{{1}} } \right)/{\text{A}}_{0} } \right] \, \times { 1}00$$

where: A_0_ = Absorbance of control, A_1_ = Absorbance of sample.

#### Hydroxyl radical (OH^.^) scavenging activity

The ability of oxyhemoglobin and methemoglobin to produce hydroxyl radicals (OH.) from hydrogen peroxides H_2_O_2_ has been studied using deoxyribose and phenylalanine as OH "detector molecules"^37^^.^ The high level of H_2_O_2_ breakdown methaemoglobin, and liberate iron ions that react with H_2_O_2_ to form a species that appears to be OH^38^. Radicles without oxygen possess one or more unpaired electrons, which makes them unstable. Hydroxyl radicals and peroxynitrite radicals are the most damaging ROS in any biological system. The breakdown of H_2_O_2_ produces primarily hydroxyl radicals^37^. Another troubling element of the hydroxyl radical is that whereas ROS species such as superoxide can be quenched by superoxide dismutase, no enzyme exists in living systems to quench the hydroxyl radical^37^. In living organisms there are two major ROS species, superoxide radicals and hydroxyl radicals continuously produce through a process of reduction of oxygen to water^35^. Moreover, hydroxyl radicals are especially harmful because of their ability to reduce disulfide bonds in proteins, and destroy fibrinogen, as a result, unfolding and unnatural refolding into the abnormal configuration of proteins^39^. Hydroxyl radicles generate in an *in-vivo* environment due to hypoxic conditions^40^. Most notably the hydroxyl radicals are produced from the decomposition of hydrogen peroxides (ROOH) is one of the most reactive forms of oxygen radicle. Its half-life in the biological system is about 1 ns and shows reaction with organic molecules at rates that approach diffusion-limited, with rate constants of 10^9^–10^10^ M^−1^ s^−1^ ^16^. The green synthesized *Mentha royleana* mediated CeONPs as shown 50% degradation potential towards hydroxyl radicle and for ascorbic acid is 53% at the highest concentration 1000 µg/ml (Fig. 4d). As the concentration of CeONPs increases gradually degradation potential of nanomaterial was increased. Similarly, in another study researcher tested a model plant *Aradopsis thaliana* L. Heynh. that lack enzymatic pathways for scavenging hydroxyl radicals in case of salinity stress, and applied only CeONPs to check their scavenging potential against hydroxyl radical. According to the study's findings, CeONPs have increased K^+^ retention in leaf mesophyll, which improves plant photosynthetic performance and biomass resistance to environmental stress^41^.

#### Reducing power

The CeONPs contain transition oxidation state between Ce^+4^ and Ce^+3^^42^. The CeONPs immediately accommodate and adapt electronic configuration according to its in available medium^42^. CeONPs exhibits oxygen vacancies on their surface, these arise through the loss of oxygen or its electrons on their surface. It shows alternation between CeO_2_ and CeO_2_x amid redox reactions^34^). Green synthesized CeONPs mimic the capabilities of various antioxidant enzymes like as catalase, superoxide oxidase, and peroxide, which initiate a variety of biological consequences such as toxic towards intracellular ROS. An Interesting feature of CeONPs play both roles as oxidation and reduction catalyst, these structural properties grant CeONPs regenerative properties but it is related with the medium in which the reaction occur. Due to efficient reducing properties, the presence of any radicle species in the environment CeONPs quenches them quickly. In the particular experimental studies, green synthesized CeONPs were used as a reducing agent the results obtained for CeONPs were 21.19% scavenging potential and 37.71% for Ascorbic acid respectively (Fig. 4c). The findings obtained from CeONPs were least near ascorbic acid but were effective in quenching radicles as CeONPs concentrations increased. Similar results were observed for another study in which CeONPs were used as reducing material within the FRAP level following the administration of CeONPs and reduction potential increased as the treatments of CeONPs were increased^43^. Another study explains the antioxidant power of CeONPs to reduce the toxicity of Malathion (which is a common pesticide in agriculture) which is tested on rays and drug is responsible to cause testicular toxicity and lower sperm count. CeONPs were found to have a lower toxic and antioxidant effect, as well as improved protection against male rat infertility^14^.

### Hypoglycemic potential of CeONPs

#### Glucose uptake by yeast cells

**Figure 5:**
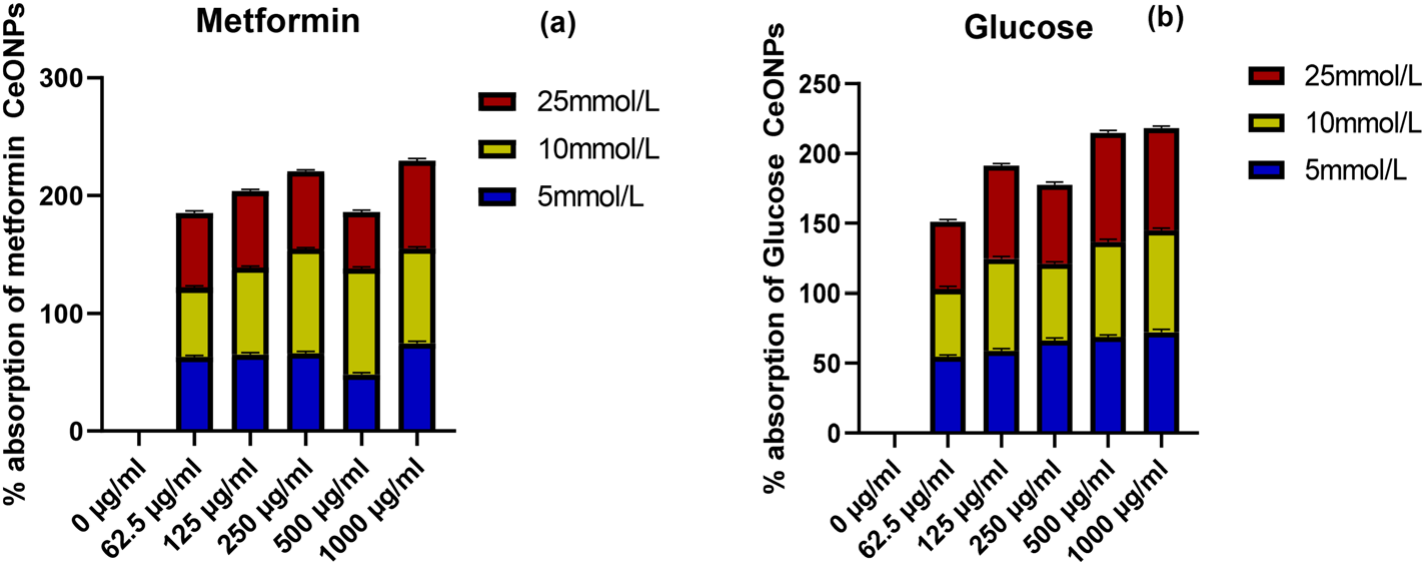
(**a**) explains the standard metformin effects on glucose absorption. The graph (**b**) explain the graphical percentage of CeONPs with three different concentrations 5, 10, and 25 mmol/L and The level of significance was *p* < 0.05 for the alpha-amylase assay. p-value lesser than 0.05 was considered significant along with the post hoc test.

Insulin's role is to absorb glucose from the blood and store it as glycogen in muscle and liver cells. During glycolysis, glucose is broken down to generate ATP (cellular work currency), and the residual glucose is transformed into fat to act as energy storage. In a present assay The oxidation of pancreatic cells and higher glucose uptake through diet raise the sugar level in the blood and lower insulin content fail to absorb glucose into liver cells and result in hyperglycemia The oxidation of pancreatic cells and increased glucose uptake via meal elevate blood sugar levels, but reduced insulin content fails to absorb glucose into liver cells, resulting in hyperglycemia^2,43^. The various types of medications are currently available on the market for hyperglycemia but the continuous increase in diabetic patients encourages researchers to explore new ways to combat diabetes. The green synthesized CeONPs are used to portrayal the insulin mechanism by binding the additional glucose from the available medium and fetching or moving the glucose into the yeast cells. The metformin was standard medicine, which is emphatically well–known for hyperglycemia. Three different concentrations of glucose were used (5, 10, 25 mmol/L) and at 1000 µg/ml for 25 mmol/L of glucose solution, *Mentha royleana* mediated CeONPs have shown 76% absorption of glucose. For 5 and 10 mmol/L of glucose concentration CeONPs has shown 72% glucose absorbance in yeast cells (Fig. 5b). Metformin was used as a standard drug famous in the market against hyperglycemia (Fig. 5a). The glucose absorption percentage for the standard was 74%, 80%, and 85% for 5, 10, 25 mmol/L gradually. As the concentrations of glucose in the solution were increased, the affinity of CeONPs for glucose was also increased. Overall, *M. royleana*-mediated CeONPs have a high affinity for glucose molecules, which is similar to metformin. Similarly, CeONPs derived from *Stachys japonica* Miq. leaf extract was discovered to be useful in regulating glucose metabolism as well as its underlying molecular mechanism for beneficial therapy against hyperglycemia^12,12^.

#### Alpha-amylase inhibition assay

**Figure 6:**
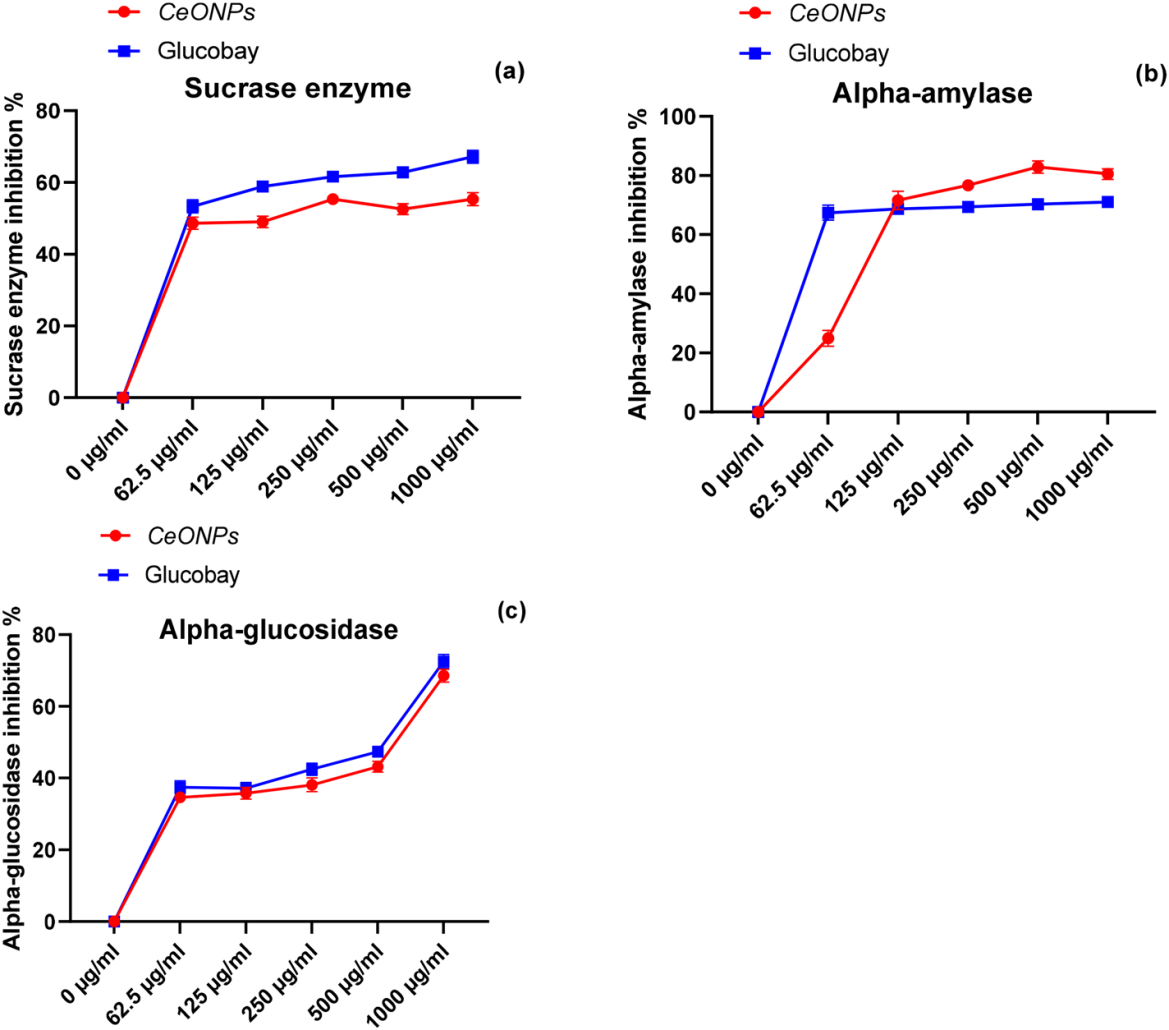
Image (**a**) show the inhibition percentage of sucrase enzyme and standard glucobay (**b**) reduction percentage of alpha-amylase and glucobay comparison (**c**) inhibitory activity of CeONPs and Glucobay against alpha-glucosidase. The statistical analysis value for level of significance was *p* < 0.05 for all anti-enzyme assays. A *p*-value lesser than 0.05 was consider significant along with the post hoc test.

All animals, including humans, have alpha-amylase in their saliva. This enzyme is a vital component of the digestive system that is secreted by the salivary glands, with a working pH of 6.7–7.0^32,39^. Alpha-amylase is an intestinal enzyme that breaks down polysaccharide -1,4 glycosidic bonds into monosaccharides for easy absorption into the blood^39^. Salivary amylase is the first step in the chemical digestion of food material. In the given assay suppression of the catalytic activity of alpha-amylase through CeONPs was checked to reduce the hydrolysis of polysaccharides, resulting low amount of glucose liberated into the blood system^40^. The metallic CeONPs bind the catalytic site of the enzyme and prevent the binding of substrate for the initiation of catalytic activity and lessen the broken-down of sugar compounds. Through the *in-vitro* procedure, green synthesized CeONPs was investigated against alpha-amylase at various concentrations ranging from 62.5 to 1000 µg/ml. In a dose-dependent manner, CeONPs have shown effectual and potent inhibition of 77.77% against α-amylase and the incubation of solution in a boiling water bath turn reduced sugar into brown–red color. The glucobay is known as modish and a well-known medicine against hyperglycemia was used as a standard drug in a given exploratory study. The proportion of sugar decrease for glucobay was 71% percent, which is much less than CeONPs (Fig. 6b). To strengthen our findings with a previously published paper, *Cassia angustifolia* leaves extract mediated CeONPs were used as an obstruct and trammel weapon against alpha- amylase, and the inhibitory value IC_50_ was found to be 89.96 µg/ml.^44^.

#### Alpha-glucosidase Inhibition Assay

**Figure 7:**
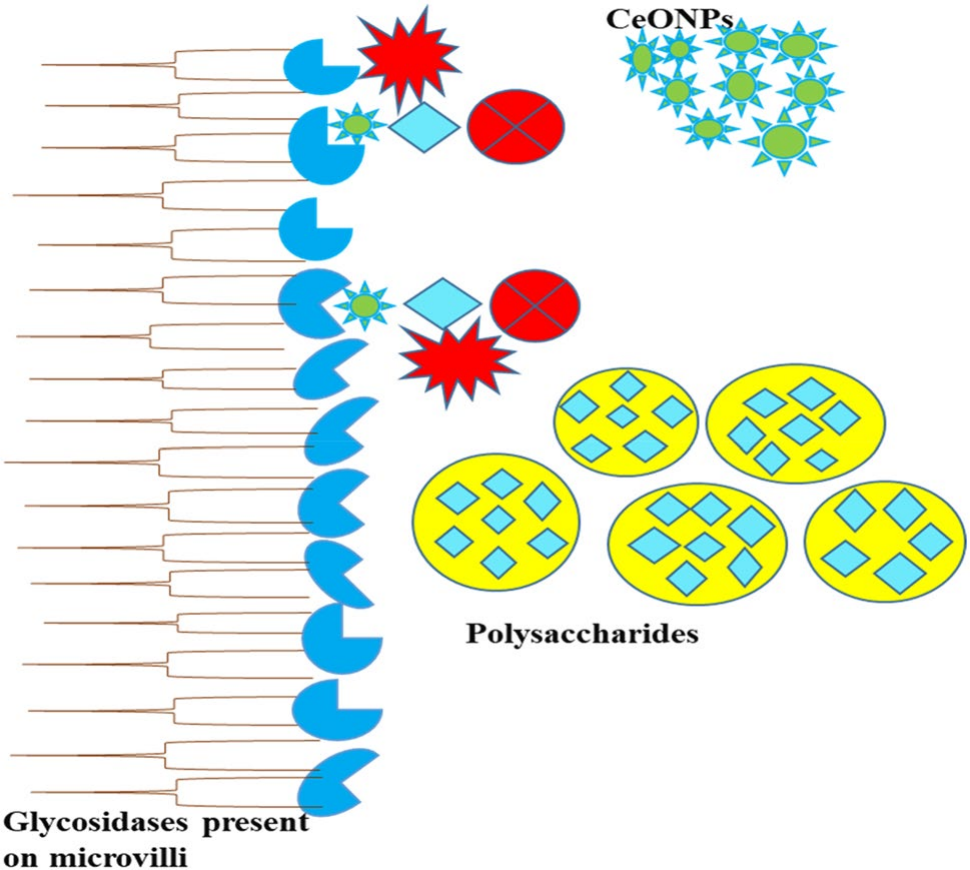
Schematic representation of inhibition potential of CeONPs against alpha-glucosidases. The alpha-glucosidase is naturally present in the microvilli in the small intestine and accelerates the breakdown of polysaccharides. CeONPs reduce the formation of the enzyme–substrate complex by covering the active site of the enzyme. The reduction of metabolic activity declines the release of glucose in the blood^12,12^.

The alpha-glucosidases use the hydrolysis of dietary cellulose, carbohydrates, and starch to convert polysaccharides into monosaccharides, which are then converted into usable glucose^45^. Glucose absorbs through the small intestine into blood vessels^46^. Alpha-glucosidases bring about the hydrolysis of terminal, alpha-1,4-linkages of various carbohydrate residues (Fig. 7). The alpha-glucosidases also called maltases. Alpha-glucosidase produce in the small intestine and positioned of the microvilli of small intestine and catalyzes the alpha (1 → 4) bonds of polysaccharides and liberates monosaccharides I environment^46^. In the current work, green synthesized CeONPs were utilized as an alpha-glucosidase inhibitor to inhibit enzyme activity and minimize polysaccharide hydrolysis, which indirectly regulated blood sugar levels. According to the results of the present research work, green synthesized CeONPs have shown 68.6% inhibition of alpha-glucosidase, and the standard glucobay a market famous drug for hypoglycemia has shown 72.6% inhibition at 1000 µg/ml concentration. For the 500 µg/ml concentration, enzyme inhibition was 82% percent for green synthesized CeONPs and 42% for glucobay, which is effective in comparison to the standard (Fig. 6c). The inhibitory percentage results of green synthesized CeO_2_NPs is very close to the output of standard medicine. Few similar examples are proposed to support our research objectives, such as *Stachys japonica* leaves extract manufactured CeONPs were investigated for their antioxidant and antidiabetic activities. Even at low concentrations, CeONPs showed significant inhibition of alpha-glucosidase^12^. Similarly *Cassia* *angustifolia* (Senna) mediated green synthesized CeONPs parade visible reduction in alpha-glucosidase catalyzing activity. The findings are plausible and well-supported for commercial uses of green synthesized CeONPs in the pharmaceutical industry^44^. In another study *Crinum viviparum* flower extract based δ-Bi_2_O_3_ were tested against New Delhi metallo-β-lactamase 1 (NDM-1) enzyme that is important because compel and enhance bacterial resistant against broad range of beta-lactam antibiotics δ-Bi_2_O_3_ NPs were tested as (NDM-1) enzyme inhibitor, and molecular docking study revealed that δ-Bi_2_O_3_ NPs showed good interaction with various amino acids residues and exhibit good hydrogen bonding^47^.

#### Sucrase inhibition assay

Every day, hundreds of enzymes perform numerous roles in the human body. Each enzyme plays a unique role in managing cellular metabolism. Sucrase is a key enzyme in the hydrolysis of sucrose (table sugar), a significant component of our daily diet^45^. The sucrase break alpha 1–4 glycosidic bond and release simple glucose and fructose molecules in the body which are easily absorbed through microspores of microvilli^32^. The sucrase is secreted from the endpoints or head of the microvilli of the epithelium in the small intestine during food digestion. The breakdown of disaccharides to monosaccharides and absorption into blood rise blood glucose levels. This causes metabolic difficulties in the form of hyperglycemia^7^. The inhibition of sucrase by green synthesized CeONPs results in a reduction of sucrose catalysis, which indirectly lowers blood glucose levels^43^. At higher doses of 1000 µg/ml CeONPs, the inhibitory potential of green synthesized CeONPs was 55% and that of standard Glucobay was 67% respectively (Fig. 6a). CeONPs' inhibitory power was seen to grip the enzyme active site and cause it to lose its catalytic function. Several studies have been reported for NPs exhibiting sucrase activity, but unfortunately, no such reports were published on the CeONPs sucrase activity. Different species i.e. *Azadirachta indica, Calotropis procera, Cephalandra indica*, *Syzygium jambolanum* were combined to use for the synthesis of gold and silver and gold NPs and these NPs were checked to reduce the catalytic power of sucrase enzyme to decline the hydrolysis of sucrose. The results obtained were noticeably exhibiting a record inhibition of sucrase activity^48^.

## Conclusion

This study investigates the biocompatibility, bioavailability, and biodegradability of non-toxic CeONPs in the presence of oxidative stress and hypoglycemia. Various characterization data show that CeONPs are 35 nm in size, circular in shape, and have a PDI of 0.2, making the CeONPs more suitable for biological applications. This research touches on both the fields of nanotechnology and phytochemistry. The applications of *Mentha royleana* fabricated green synthesized CeONPs showed maximum antioxidant and hypoglycemic potential. The response of CeONPs is dose-dependent, with 500 µg/ml and 1000 µg/ml being proven to be effective doses. The findings of the hydrogen peroxide scavenging assay and the hydroxyl radical scavenging assay were higher than those of ascorbic acid and DPPH, whereas the results of ABTS^+^ and reducing power were closer to the standard. CeONPs inhibited alpha-amylase more effectively than Glucobay in antienzyme assay. The inhibitory potential of alpha-glucosidase, antisucrase, and glucose absorption by yeast cells was comparable to that of glucobay and metformin. *Mentha royleana* phyto-constituents may play a synergistic role in improving antioxidant and hypoglycemic characteristics CeONPs. In-vitro assay results explain the anti-diabetic and antioxidant effect of green synthesized CeONPs for future in-vivo and clinical investigations, as well as drug manufacturing^49–52^.

## Data Availability

The datasets used and/or analyzed during the current study available from the corresponding author on reasonable request.
